# Stop wasting protein—Proteasome inhibition to target diseases linked to mitochondrial import

**DOI:** 10.15252/emmm.201910441

**Published:** 2019-04-03

**Authors:** Markus Habich, Jan Riemer

**Affiliations:** ^1^ Department of Chemistry Institute for Biochemistry University of Cologne Cologne Germany

**Keywords:** Genetics, Gene Therapy & Genetic Disease, Pharmacology & Drug Discovery

## Abstract

Mitochondrial dysfunction is linked to various human diseases. Symptoms can occur early in life or manifest progressively during life and include poor muscle coordination or weakness, neurological or developmental problems, or immunodeficiency (Lightowlers *et al*, 2015). Most mitochondrial diseases are caused by mutations in genes encoding mitochondrial proteins. Mutations can affect protein functions in many ways; they can not only impair enzymatic activities, but also lower protein stability, hamper assembly into multimeric protein complexes, or abrogate protein transport into mitochondria. Understanding the impact of mutations on protein function is crucial to understand pathophysiological mechanisms of mitochondrial diseases and to develop therapeutic approaches.

Several disease‐related proteins are localized in the mitochondrial intermembrane space (IMS) and are subunits of respiratory chain complexes or are involved in complex assembly. The majority of these proteins is imported into the IMS via the disulfide relay machinery. This machinery handles proteins lacking N‐terminal mitochondrial targeting signals (MTS) and instead employs oxidation of conserved cysteines for import. Import by this machinery appears to be slow compared to MTS‐dependent pathways, which allows for competition of mitochondrial import with cytosolic degradation. Consequently, mitochondrial levels of at least some disulfide relay substrates are controlled by the ubiquitin–proteasome system (UPS). Classical substrates of this pathway are small proteins that share characteristic folds and cysteine patterns. Recently, several non‐conventional substrates were identified that do not fit these features demonstrating that the disulfide relay possesses a high degree of substrate versatility (Habich *et al*, 2019b).

In the current issue of *EMBO Molecular Medicine*, Mohanraj *et al* (2019) identified cytochrome *c* oxidase assembly factor 7 (COA7) as a novel non‐conventional disulfide relay substrate. Cytochrome *c* oxidase assembly factor 7 is involved in the assembly of complex IV (Kozjak‐Pavlovic *et al*, 2014). It was found to be mutated in a patient who presented with mitochondrial leukoencephalopathy and complex IV deficiency (Martinez Lyons *et al*, 2016). Mohanraj *et al* demonstrate that disease‐causing COA7 variants were affected in their mitochondrial import and were thus present in decreased amounts in patient fibroblasts. Instead of being imported, disease‐causing COA7 variants became degraded in the cytosol via the UPS. COA7 variants were not completely impaired in IMS import and folding, but their import and folding was strongly slowed down. Preventing cytosolic degradation via pharmacological inhibition of the proteasome thus not only increased cellular COA7 mutant levels, but also enabled mitochondrial import of COA7 variants, and restored complex IV activity and supercomplex formation (Mohanraj *et al*, 2019; Fig**** 1).

**Figure 1:**
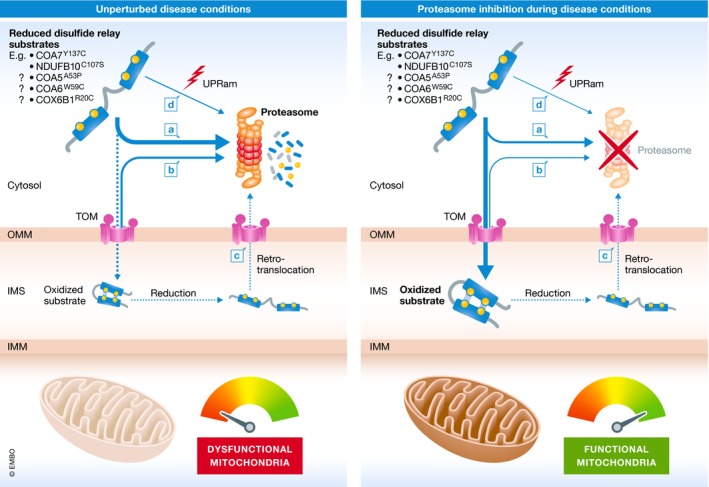
The proteasome controls disulfide relay‐mediated protein import at different stages Disease variants are preferentially targeted for proteasomal degradation resulting in lower levels of these proteins in mitochondria and mitochondrial dysfunction. Proteasomal inhibition allows increased accumulation of disease variants in mitochondria resulting in improved mitochondrial function.

At which stage during import and folding might modulation of UPS activity affect mutant protein maturation? The UPS has been linked to mitochondrial import by the disulfide relay machinery at multiple stages (Fig**** 1). It is involved in controlling levels of IMS proteins by degrading precursors while they are *en route* to mitochondria (Bragoszewski *et al*, 2013; Fig 1A). This might be an important regulatory step for mitochondrial biogenesis especially during changes in nutrient supply. UPS inhibition increases amounts of available mutated precursor proteins in the cytosol. This increases the likelihood for import even for mutated substrates, which are imported only very slowly. The UPS also serves as part of a redox quality control pathway, in which precursors are tested for import and folding competence by the disulfide relay machinery itself (Fig 1B). Translocating substrates that are rejected by the disulfide relay slide back to the cytosol where they become degraded (Habich *et al*, 2019a). UPS inhibition would prevent removal of substrates, which failed this quality control step. It would thereby enable their full translocation into the IMS, proper folding and function. The UPS also monitors the levels of mature IMS proteins. It is part of a degradation pathway in which proteins are reduced in the IMS and then retrotranslocated for cytosolic degradation (Bragoszewski *et al*, 2015; Fig 1C). UPS inhibition might stabilize mature proteins that otherwise would be retrotranslocated and degraded in the cytosol, thereby limiting their turnover and contributing to increased steady‐state levels. Lastly, the UPS is an important part of stress response pathways (UPRam) in which it partakes in clearing precursors that accumulate in the cytosol if mitochondrial import *per se* is impaired (Wrobel *et al*, 2015; Fig 1D). Thus, cytosolic accumulation of mutated precursor proteins could result in the secondary loss of other mitochondrial proteins, which might be prevented by attenuation of proteasomal activity.

The findings by Mohanraj and colleagues have implications beyond COA7. Point mutations in various disulfide relay substrates have been linked to severe human disease. These include mutations in NDUFB10 (C107S), TIMM8A (C66W), COA5 (A53P), COA6 (W59C), and COX6B1 (R20C) that likely all affect IMS import and folding (see For more information). For NDUFB10^C107S^, an interesting tissue heterogeneity was observed; low levels of NDUFB10 and consequently low complex I activity were detected in patient liver and muscle, but almost normal protein levels and complex I activity were found in skin (Friederich *et al*, 2017). This strongly suggests that mutated NDUFB10 can in principle be imported, folded, and assembled into complex I but fails to do so in most tissues. UPS inhibition stabilizes mutant NDUFB10 (Habich *et al*, 2019a) and therefore may as well constitute a therapeutic strategy to enable mitochondrial accumulation in this disorder.

The proteasomal inhibitors bortezomib and carfilzomib, which were used by Mohanraj and colleagues, are already clinically approved for the treatment of patients with multiple myeloma and mantle cell lymphoma. Although these inhibitors have been associated with side effects such as cardiotoxicity and peripheral neuropathy (Kaplan *et al*, 2017), the severity of mitochondrial diseases might justify their employment. Given the necessary long‐term treatment of patients suffering from mitochondrial diseases, a major challenge might lie in the acquisition of resistance against proteasome inhibitors. Tumor cells were shown to acquire resistance against proteasome inhibitors by, e.g., upregulating proteasome biogenesis. A possible solution to overcome this problem might therefore be to apply combination therapies using proteasome inhibitors and molecules interfering with proteasome biogenesis. Collectively, Mohanraj and colleagues introduce proteasomal inhibition as a novel concept to treat mitochondrial diseases that are caused by protein import defects.

**For more information**

(i) COA7 (Y137C): https://www.omim.org/entry/615623#0001
(ii) NDUFB10 (C107S): https://www.omim.org/entry/603843?search=ndufb10&highlight=ndufb10 and (Friederich *et al*, 2017)
(iii) TIMM8A (C66W): https://www.omim.org/entry/300356#0004
(iv) COA5 (A53P): https://www.omim.org/entry/613920#0001
(v) COA6 (W59C): https://www.omim.org/entry/614772#0001
(vi) COX6B1 (R20C): https://www.omim.org/entry/124089#0002
